# Prenatal Exposure to Lipopolysaccharide Results in Local RAS Activation in the Adipose Tissue of Rat Offspring

**DOI:** 10.1371/journal.pone.0111376

**Published:** 2014-10-31

**Authors:** Meng Gao, Xingxing Zhang, Xin Chen, Cunyun Mi, Yujie Tang, Jianzhi Zhou, Xiaohui Li

**Affiliations:** 1 Institute of Materia Medical, College of Pharmacy, Third Military Medical University, Chongqing, China; 2 Department of Medicament and Instrument, 159 Hospital of PLA, Zhumadian, Henan, China; Max-Delbrück Center for Molecular Medicine (MDC), Germany

## Abstract

**Background:**

Adult metabolic syndrome may originate in part during fetal or early life. This study was designed to investigate the effects of prenatal exposure to lipopolysaccharide (LPS) on adipose development and local renin-angiotensin system (RAS) activation in rat offspring.

**Methods:**

Pregnant rats were randomly divided into three groups (n = 8 in each), including an NS group (pregnant rats were only treated with 0.5 ml normal saline from the 8^th^ to the 14^th^ day of gestation); an LPS group (pregnant rats were injected intraperitoneally with 0.79 mg/kg LPS on the 8^th^, 10^th^ and 12^th^ days of pregnancy); and an LPS+pyrrolidine dithiocarbamate (PDTC) group (identical to the LPS group except that 100 mg/kg PDTC was administered from the 8^th^ to the 14^th^ day of gestation).

**Results:**

Prenatal exposure to LPS resulted in increased blood pressure, adipose coefficient and body weight in rat offspring. Specifically, during the infancy of the offspring rats, the LPS stimulus promoted the differentiation of adipose cells, diminishing their diameters and proportions while simultaneously increasing cell number. In contrast, once the rats were grown, adipose cell differentiation was inhibited, and the diameters and proportions of the cells were increased. Moreover, each component of the RAS was changed and was shown to be activated. PDTC, an inhibitor of NF-κB, could reverse the influence of the stimulus during pregnancy.

**Conclusion:**

Prenatal exposure to LPS in rats results in increased blood pressure, adipose coefficient, body weight and activation of adipose RAS in offspring.

## Introduction

Essential hypertension (EH) is the leading risk factor for cardiovascular disease, and it is a serious threat to human health [1]. The present study demonstrates that fetal environmental factors during pregnancy are closely related to adult diseases (hypertension, diabetes and obesity) [2]. This idea has also been supported by global epidemiological investigations and experimentation [3]. Previous work from our group has also shown that inflammatory immune stimulation in pregnant rats results in hypertension, increased leptin levels and increased body weight and fat tissue weight in offspring [4], [5]. The circulating renin-angiotensin system (RAS) of offspring rats with hypertension does not significantly changes, but there is a significant rise in angiotensin II (Ang II) expression in kidney tissue [6], [7]. These results suggest that inflammatory stimulation during pregnancy affects fat metabolism in offspring rats. At the same time, the change in local tissue RAS may be an important mechanism connected with hypertension [8].

Adipose tissue can secrete a variety of autocrine- and paracrine-acting adipocytokines that can participate in regulating the RAS and in the development of fat cells themselves [9]. Notably, the local adipose tissue RAS constitutes a complete RAS that is independent of the circulating RAS. Specifically, it can play key roles in regulating adipocyte differentiation, adipocytokine production, local blood flow and blood pressure, and it can regulate target organs by autocrine or paracrine pathways [10]. Therefore, this study is focused on RAS components secreted by fat cells, as they may represent an important link between obesity and cardiovascular diseases such as hypertension [4].

On the basis of previous work, this study was designed to explore the effect of prenatal exposure to LPS (0.79 mg/kg) combined with the NF-κB inhibitor pyrrolidine dithiocarbamate (PDTC) on offspring rats. To do so, fat development in offspring rats was studied at different time points. Changes in the gene and protein expression levels of the local adipose tissue RAS were studied to explore the role and mechanism of the local adipose tissue RAS in the hypertension resulting from prenatal exposure to LPS.

## Materials and Methods

### Animals

Nulliparous, time-mated Sprague-Dawley (SD) rats were purchased from the Animal Center of the Third Military Medical University (Chongqing, China). All the rats had ad libitum access to both standard laboratory rat chow and tap water and were caged individually in a temperature-controlled room (24°C) with a 12 h/12 h light/dark cycle until parturition. Pups were raised with a lactating mother until 4 weeks of age, after which time they lived in cages with 3 rats per cage. The present study was conducted in accordance with the principles outlined in the National Institutes of Health (NIH) Guide for the Care and Use of Laboratory Animals (http://grants1.nih.gov/grants/olaw/) and was approved by the local animal ethics committee at the Third Military Medical University.

### Dams and litters

The pregnant rats were randomly divided into three groups (n = 8 in each): an NS (normal saline) group, an LPS group and an LPS+PDTC group. On the 8^th^, 10^th^ and 12^th^ days of gestation, the LPS dams (n = 8) received intraperitoneal (ip) injections of 0.79 mg/kg LPS (Sigma Chemical, St Louis, MO, USA) dissolved in 1 ml of sterile saline. The NS dams (n = 8) received 0.5 ml of sterile saline from the 8^th^ to the 14^th^ day of gestation. The LPS+PDTC dams (n = 8) were treated identically to the LPS group, except that 100 mg/kg PDTC was administered from the 8^th^ to the 14^th^ day of gestation.

### Blood pressure measurement

Systolic blood pressure (SBP) was measured in conscious offspring rats at 8, 12 and 16 weeks of age using the standard tail-cuff method (ML125, Powerlab, AD Instruments, Castle Hill, NSW, Australia). Before measurement of the SBP, rats were placed inside a warming chamber (approximately 34°C) for 15 min to calm the animals and dilate the tail blood vessels. The rats were then placed in plastic restrainers, and a cuff with a pneumatic pulse sensor was attached to the tail. In each rat, mean SBP was calculated from three consecutive SBP recordings.

### Body weight

The body weight of offspring rats from age 2 to 16 weeks was regularly monitored at 2-week intervals during the experiments.

### Adipose tissue wet weight and coefficient

The 6- and 16-week-old rat offspring were decapitated after weighing. Next, the wet weights of perirenal and mesenteric adipose tissue were measured (g). The coefficient of adipose tissue was calculated as follows: fat factor = wet weight/body weight×100.

### Adipose cell size

The same fat tissue samples from the 6- and 16-week-old offspring rats were collected and incubated in 4% paraformaldehyde solution for 48 h before being dehydrated and embedded in paraffin wax; the tissue was sliced into 4-µm sections, and HE staining was performed. Morphological changes in adipose cells were observed under a light microscope, and the number and diameter (µm) of cells were recorded.

### Oral glucose tolerance test

After fasting for 10 h, the 6- and 16-week-old offspring rats were challenged with 2 g/kg and 50% glucose solution (oral glucose tolerance test [OGTT]). Blood samples were collected at 0, 30, 60, 120 and 180 min. Glucose levels were determined using an ACCU-CHEK glucose meter (ROCHE, Germany), and we calculated the area under the curve(AUC) for glucose.

### Determination of lipid levels

Blood was obtained from the retro-orbital plexus of the eye under anesthesia and prepared for hematological and biochemical examination. Serum TC, TG, HDL-C and LDL-C were measured in the Laboratory Department of the Xinqiao Hospital (Chongqing, China).

### RT-PCR analysis of adipocyte differentiation markers

The expression levels of mRNAs encoding peroxisome proliferator-activated receptor (PPARγ), enhancer binding protein α (CEBP/α), enhancer binding protein β (CEBP/β) and transcription factor aP2 were assessed by real-time PCR when the offspring rats were 6 and 16 weeks old. Total RNA was extracted from kidneys using an RNAsimple Total RNA Kit (TIANGEN Biotech, Beijing, China) and was quantified by measuring absorbance at 260 nm. Total RNA (1 µg) was then reverse-transcribed into cDNA using a PrimeScript RT Reagent Kit with gDNA Eraser (TaKaRa Biotechnology, Dalian, China). β-Actin was used as an internal control. PCR primers were designed using Premier 5.0 (PREMIER Biosoft International, Palo Alto, CA, USA) with published nucleotide sequences. The sequences of the primers used in this study are presented in Table 1. Each real-time PCR reaction was conducted in a total volume of 25 µl with SYBR Premix Ex Taq II (Tli RNaseH Plus) (TaKaRa Biotechnology, Dalian, China) and in an Eppendorf MasterCycler ep realplex system (Eppendorf, Hamburg, Germany) under the following conditions: 30 s at 95°C and then 40 cycles at 95°C for 15 s, 60°C for 15 s and 72°C for 20 s. After amplification, a melting curve analysis was performed by collecting fluorescence data while increasing the temperature from 65°C to 99°C over a period of 135 s. The relative expression ratio of each mRNA was calculated using the equation 1/2∧ΔΔCt.

**Table 1:** RT-PCR primers and respective sequences.

Name	5-3′	Primers
aP2	FW	ATGAAAGAAGTGGGAGTTGGC
aP2	RV	CAGTTTGAAGGAAATCTCGGTGT
PPARγ	FW	ATGACAGACCTCAGGCAGATTG
PPAR*γ*	RV	TGTCAGCGACTGGGACTTTTC
CEBP/*α*	FW	TCAAGGGCTTGGCTGGTCC
CEBP/*α*	RV	CGCGATGTTGTTGCGTTCC
CEBP/*β*	FW	CACCGGGTTTCGGGACTTG
CEBP/*β*	RV	CCCGCAGGAACATCTTTAAGTG
AGT	FW	TTCAGGCCAAGACCTCCC
AGT	RV	CCAGCCGGGAGGTGCAGT
ACE	FW	CCACCGTTACCAGAC AACTATCC
ACE	RV	GCGTATTCGTTCCACAACACCT
AT1-R	FW	AA TGAGCACGCTTTCTTACCG
AT1-R	RV	AGGCTGCCCTGGCTTCTGTC
AT2-R	FW	GGAAGAACAGAATTACCCGTGACC
AT2-R	RV	CAGGAGGA TGGCAAAAGGAAGT
β-actin	FW	ACGGTCAGGTCATCACTATCG
β-actin	RV	GGCATAGAGGTCTTTACGGATG

Abbreviations: Adaptin2 (aP2); Peroxisome proliferator activator receptor gamma (PPAR *γ*); CAAT enhancer binding protein alpha (CEBP/*α*); CAAT enhancer binding protein alpha (CEBP/*β*); Angiotensinogen (AGT); Angiotenin-converting enzyme (ACE); AngiotensinII type1 receptor (AT1-R); AngiotensinII type2 receptor (AT2-R).

### Immunohistochemistry

The fat tissues from 6- and 16-week-old rat offspring were collected and incubated in 4% paraformaldehyde, embedded in paraffin, and sectioned. The sections were incubated with anti-Ang II (1∶200, Abcam, UK) for 2 h at 37°C and then with goat anti-rabbit IgG conjugate (Santa Cruz, USA) for 30 min at 37°C. After each incubation, the slides were washed 3 times with Tris-buffered saline with Tween 20 (TBST) for 5 min each. The slides were then counterstained with Mayer's hematoxylin for 15 s. When observed and photographed under the microscope, positive regions appear brown and yellow. Light micrographs were captured with a color video camera (Nikon Microscope E-100, Japan) and analyzed with image analysis software (Image Pro Plus, USA).

### Local adipose tissue RAS RT-PCR

The expression levels of mRNAs encoding renal cortex angiotensinogen (AGT), angiotensin-converting enzyme (ACE), angiotensin receptor 1 (AT_1_-R) and angiotensin receptor 1 (AT_2_-R) were assessed by real-time PCR when the offspring were 6 and 16 weeks old. Total RNA was extracted from kidneys using an RNAsimple Total RNA Kit (TIANGEN Biotech, Beijing, China) and was quantified by measuring the absorbance at 260 nm. Total RNA (1 µg) was then reverse-transcribed into cDNA using a PrimeScript RT Reagent Kit with gDNA Eraser (TaKaRa Biotechnology, Dalian, China). β-Actin was used as an internal control. The PCR reaction volume, reaction conditions and melting curve analysis were as described above. The relative expression ratio of each mRNA was calculated by the equation 1/2∧ΔΔCt.

### Western blot

Total protein in the adipose tissue of 6- and 16-week-old rat offspring was extracted, and the protein concentration was measured by the bicinchoninic acid (BCA) method. After denaturation and electrophoresis on sodium dodecyl sulfate (SDS)-polyacrylamide gels, separated proteins were transferred to nitrocellulose membranes. Membranes were then blocked in 5% nonfat milk in TBST for 1 h. After incubation with primary antibodies [anti-AT1-R (Abcam, UD), anti-AT2-R (Abcam, UK), anti-ACE (Santa Cruz, USA) or anti-β-actin (Sigma, USA)] in TBS at 4°C overnight, membranes were incubated with peroxidase-conjugated secondary antibody in TBS at room temperature for 1 h. Specific bands were detected with a chemiluminescence assay and recorded on X-ray film. The Quantity One software (Bio-Rad, USA) was used to quantify the band intensities.

### Statistical analysis

Results are presented as means ± SD. Comparisons among groups were made using one-way ANOVA, and P<0.05 was considered significant. All analyses were performed with SPSS 13.0 (SPSS Inc. Chicago, IL, USA).

## Results

### Blood pressure measurement

**Figure 1:**
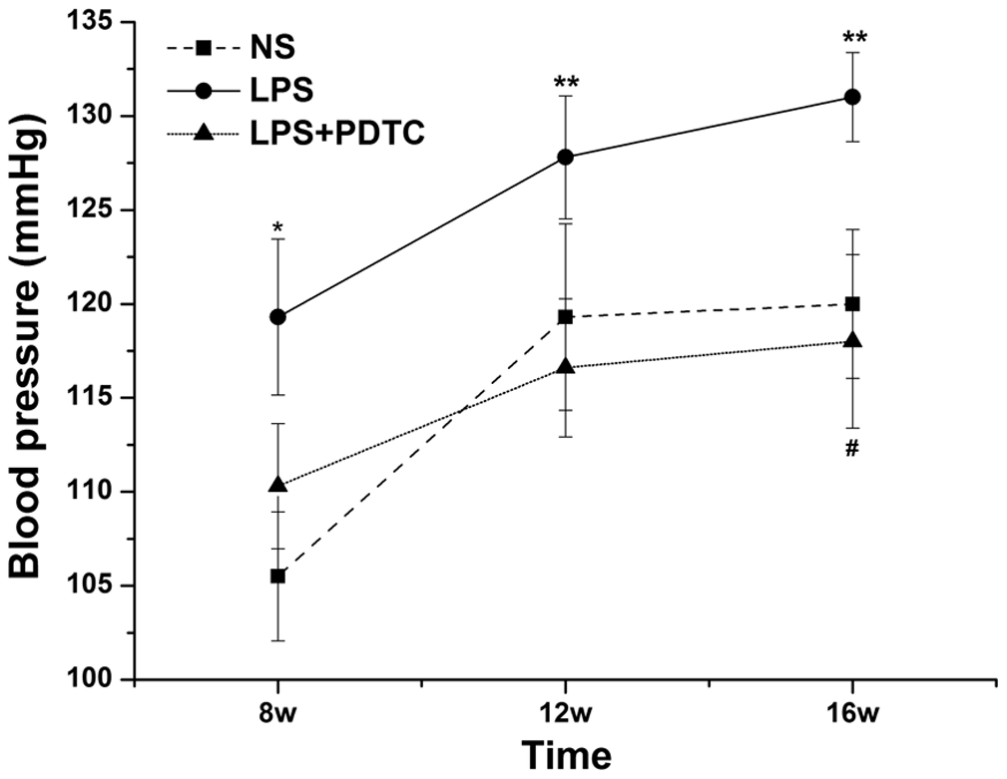
The effects of prenatal exposure to LPS or LPS+PDTC on blood pressure in rat offspring. n = 12. Values represent mean ± SD.*p<0.05, **p<0.01 vs. NS; #*p*<0.05, ##*p*<0.01 vs. LPS.

All offspring with prenatal exposure to LPS had elevated SBP at 6 and 16 weeks of age. SPB in the LPS group increased significantly compared with the NS group at 6 weeks of age (119.3±4.15 mm Hg *vs.* 105.5±3.43 mm Hg, *P*<0.05; 1 mm Hg = 0.133 kPa). At 16 weeks of age, the difference between these 2 groups became more significant (131.0±2.37 mm Hg *vs.* 120.0±3.96 mm Hg, *P*<0.01). In addition, SBP was significantly decreased in the LPS+PDTC group compared with the LPS group (118.0±4.62 mm Hg *vs.* 131.0±2.37 mm Hg, P<0.05) (Figure 1).

### Body weight

**Figure 2:**
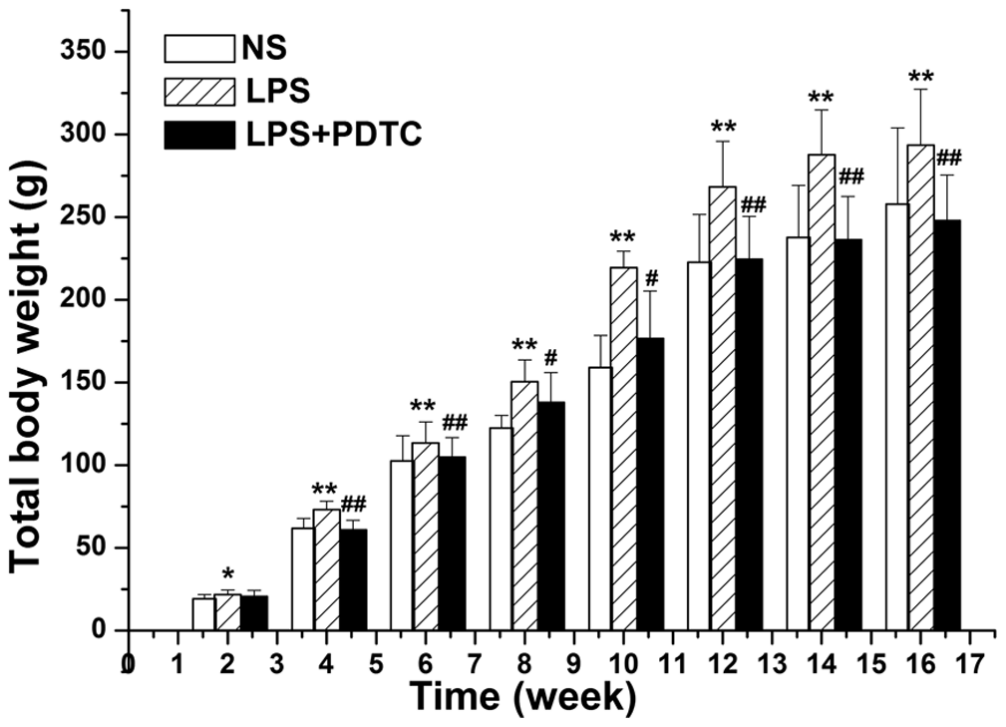
Total body weight (g) at 2 to 16 weeks of age. n = 12. Values represent mean ± SD.*p<0.05, **p<0.01 vs. NS; #*p*<0.05, ##*p*<0.01 vs. LPS.

The body weight of rat offspring from 2 to 16 weeks of age was measured once per week, and the mean body weight of offspring in the LPS group was significantly higher than that of the control group or the LPS+PDTC group. In the offspring, the difference in total body weight between the LPS and control groups reached statistical significance by 2 weeks of age (21.81±2.75 g *vs.* 19.26±2.53 g, *P*<0.05). However, the difference became more statistically significant by 16 weeks of age (293.61±33.67 g *vs.* 257.78±46.12 g, *P*<0.01). At 16 weeks of age, the body weight of the LPS+PDTC group was significantly decreased compared with that of the LPS group (247.87±27.52 g *vs.* 293.61±33.67 g, *P*<0.01) (Figure 2).

### Adipose tissue wet weight and coefficient

**Figure 3:**
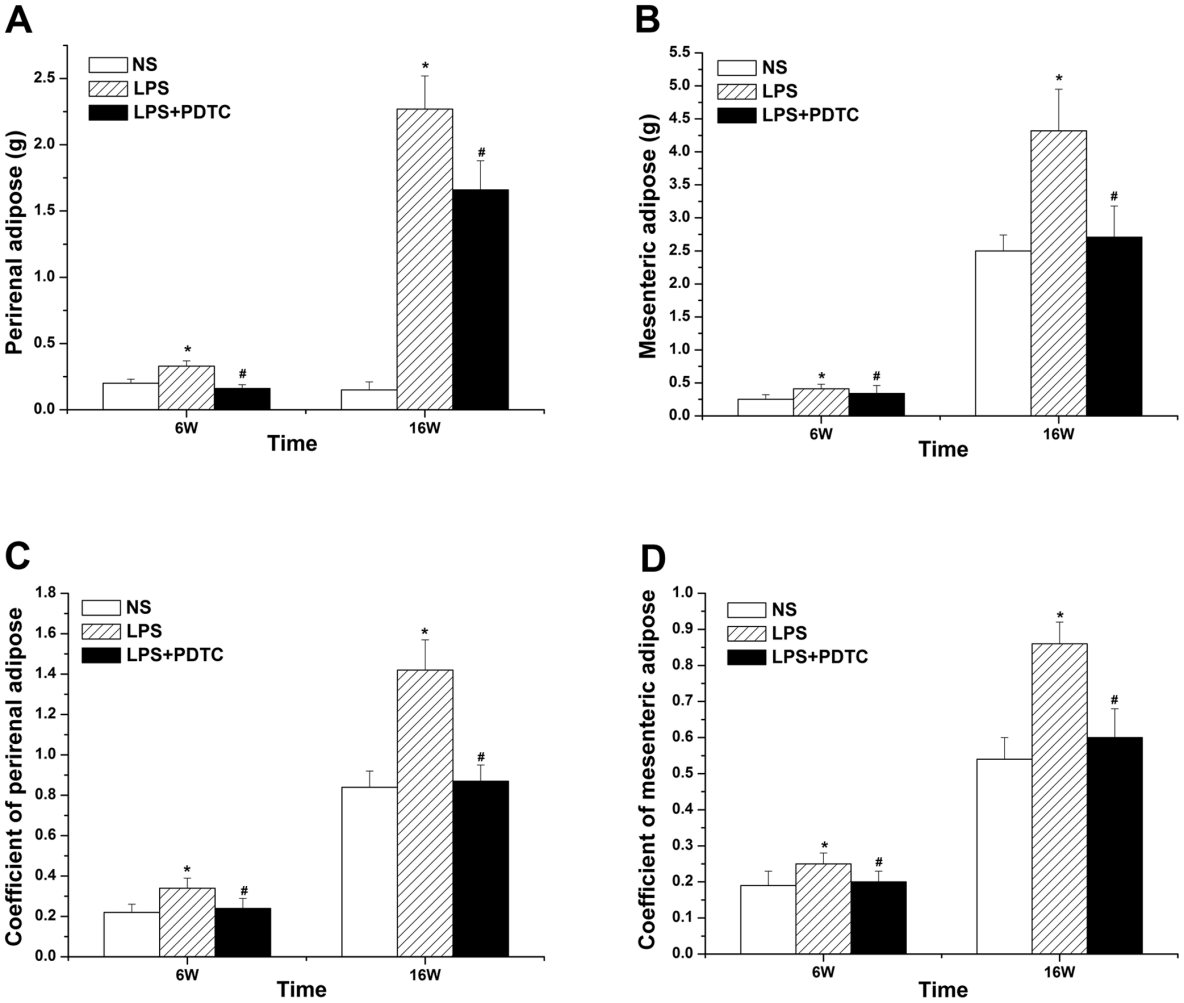
The effects of prenatal exposure to LPS/LPS+PDTC on the wet weight of perirenal adipose tissue (A), the wet weight of mesenteric adipose tissue (B), the coefficient of perirenal adiposity (C), and the coefficient of mesenteric adiposity (D) in 6- and 16-week-old offspring rats. n = 8. Values represent mean ± SD. *p<0.05, **p<0.01 vs. NS; #*p*<0.05, ##*p*<0.01 vs. LPS.

The wet weight and adipose coefficient of perirenal and mesenteric adipose tissue in the LPS group prominently increased (*p*<0.05) compared with the NS group, whereas the value in the LPS+PDTC group decreased significantly (*p*<0.05) compared with the LPS group. Both 6- and 16-week-old offspring showed the same trend, and all of the results were significantly different (P<0.05) (Figure 3).

### Adipose cell size

**Figure 4:**
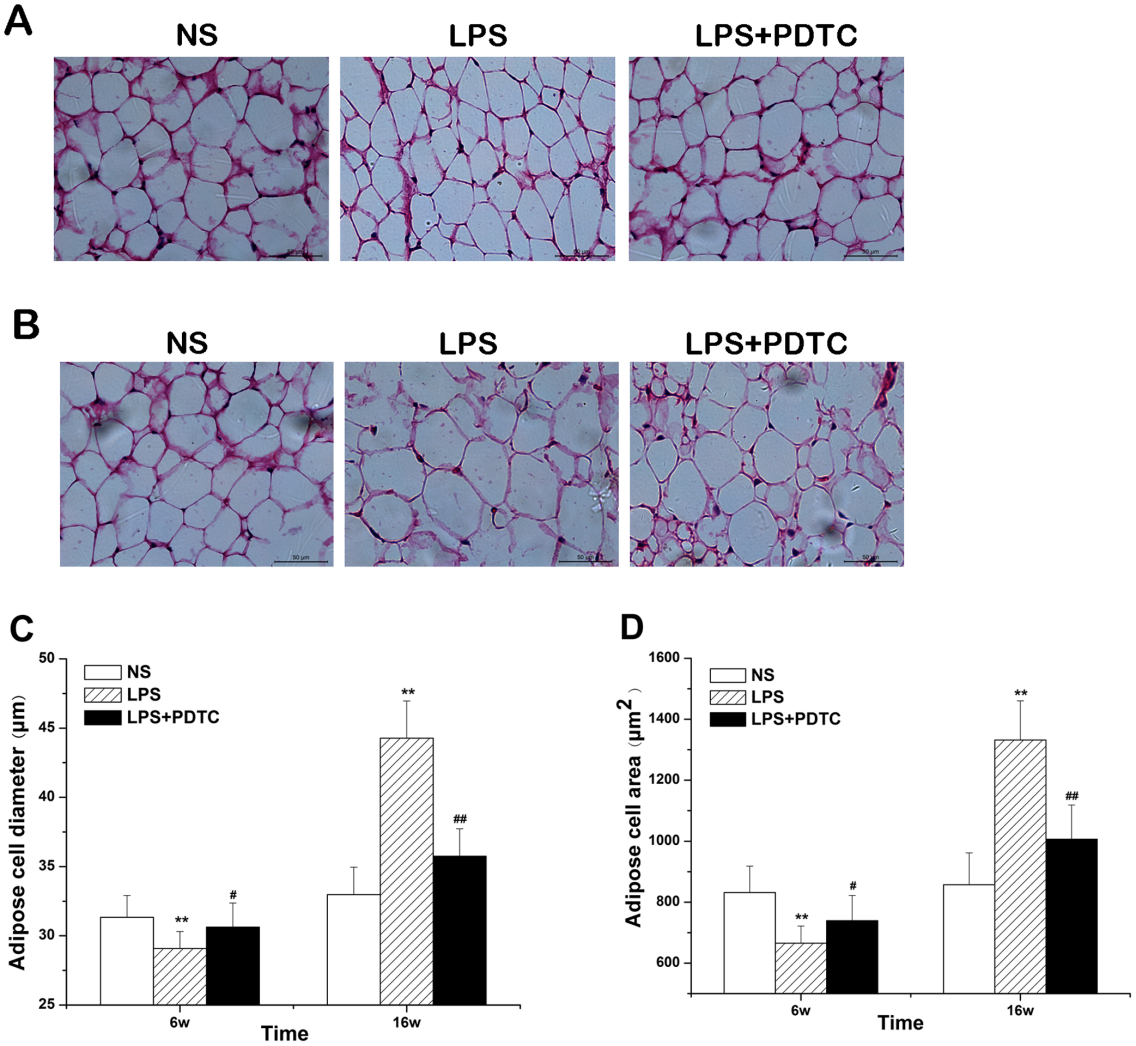
Prenatal exposure to LPS alters of adipose cell size in 6 (A) and 16-week-old (B) offspring rats (HE staining). The effects of prenatal exposure to LPS/LPS+PDTC on adipose cell diameter (C) and adipose cell area (D) in offspring. n = 8. Values represent mean ± SD. *p<0.05, **p<0.01 vs. NS; #*p*<0.05 vs. LPS.

The mean adipose cell diameter in offspring in the LPS group was significantly lower than that in the NS group (29.08±1.23 µm *vs.* 31.33±1.59 µm, *P<*0.01) or in the LPS+PDTC group (29.08±1.23 µm *vs.* 30.63±1.74 µm, *P*<0.05) at 6 weeks of age. In contrast, at 16 weeks of age, the mean adipose cell diameter in offspring in the LPS group was strikingly higher than that in the NS group (44.27±2.69 µm *vs.* 32.97±1.99 µm, *P*<0.01) or in the LPS+PDTC group (44.27±2.69 µm *vs.* 35.75±1.98 µm, *P*<0.01) (Figure 4).

### OGTT and lipid levels

**Figure 5:**
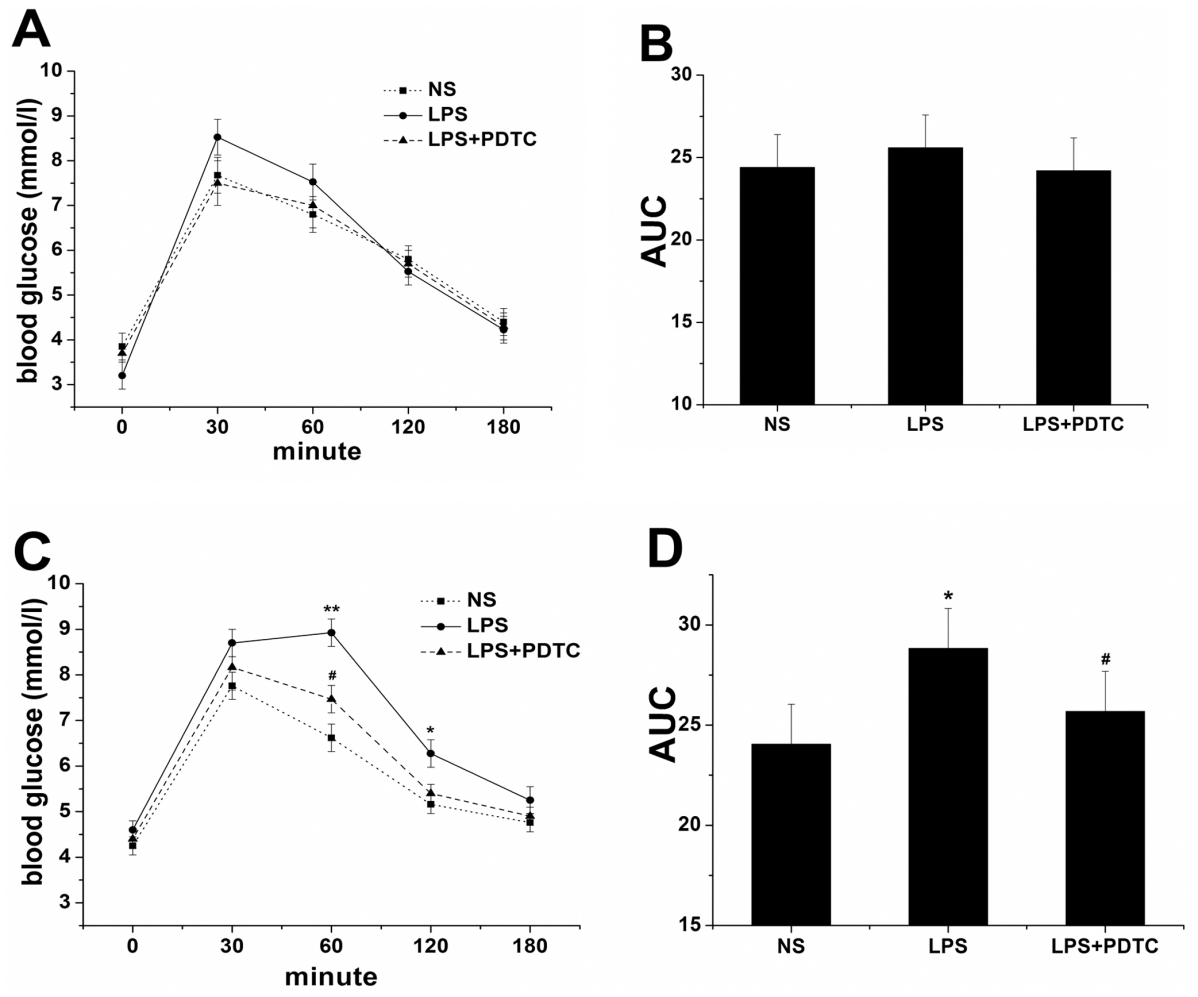
The effects of prenatal exposure to LPS/LPS+PDTC on OGTT in 6- (A) and 16-week-old (C) offspring rats. Glucose AUC results in 6- (B) and 16-week-old (D) offspring rats. n = 8. Values represent mean ± SD. *p<0.05, **p<0.01 vs. NS; #*p*<0.05 vs. LPS.

**Figure 6:**
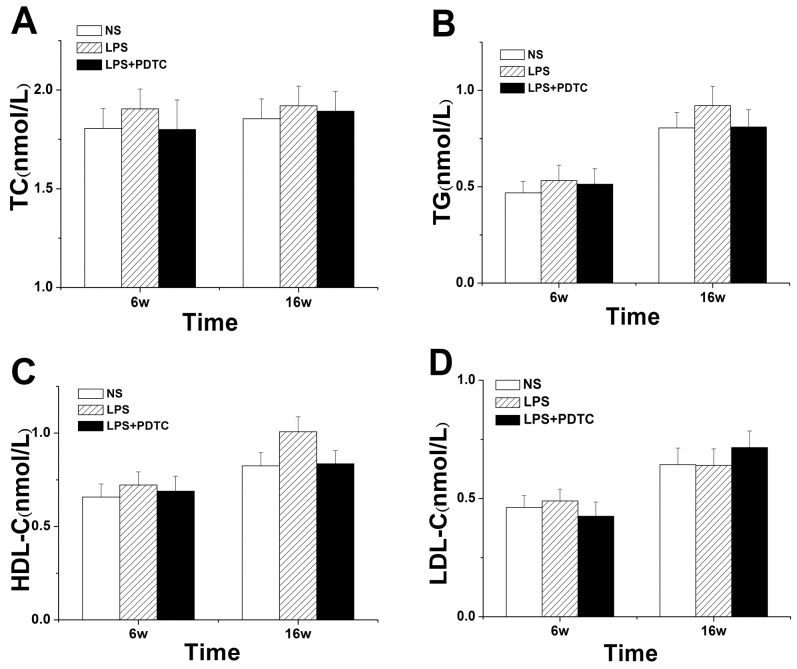
Prenatal exposure to LPS combined with PDTC alters TC (A), TG (B), HDL-C (C) and LDL-C (D) expression in rat offspring. n = 8. Values represent mean ± SD. *p<0.05, **p<0.01 vs. NS; #*p*<0.05 vs. LPS.

Fasting glucose levels did not significantly differ among the three groups in 6 and 16-week-old offspring. During the OGTT, glucose levels peaked at 30 min and then gradually returned to baseline by 180 min in the three groups of 6-week-old offspring (Figure 5A). The glucose AUC exhibited the same trend (Figure 5B). At 16 weeks, glucose levels peaked at 30 min in the NS and LPS+PDTC groups, whereas the levels, peaked at 60 min in the LPS groups. Moreover, glucose levels in the LPS group were significantly increased compared with the NS (8.93±0.66 mmol/L *vs.* 6.62±0.52 mmol/L, *P<*0.01) and LPS+PDTC groups (8.93±0.66 mmol/L *vs.* 7.47±0.61 mmol/L, *P*<0.05) at 60 min. In addition, glucose levels in the LPS group were significantly increased compared with the NS group (6.28±0.43 mmol/L *vs.* 5.16±0.34 mmol/L, *P*<0.05) at 120 min (Figure 5C). Similarly, the glucose AUC in 16-week-old rat offspring was strikingly increased in the LPS group compared with the NS(*P*<0.05) and LPS+PDTC groups (*P*<0.05) (Figure 5D). However, TC, TG, HDL-C and LDL-C levels did not significantly differ among the groups at 6 and 16 weeks (*P*>0.05) (Figure 6).

### RT-PCR analysis of adipocyte differentiation markers

**Figure 7:**
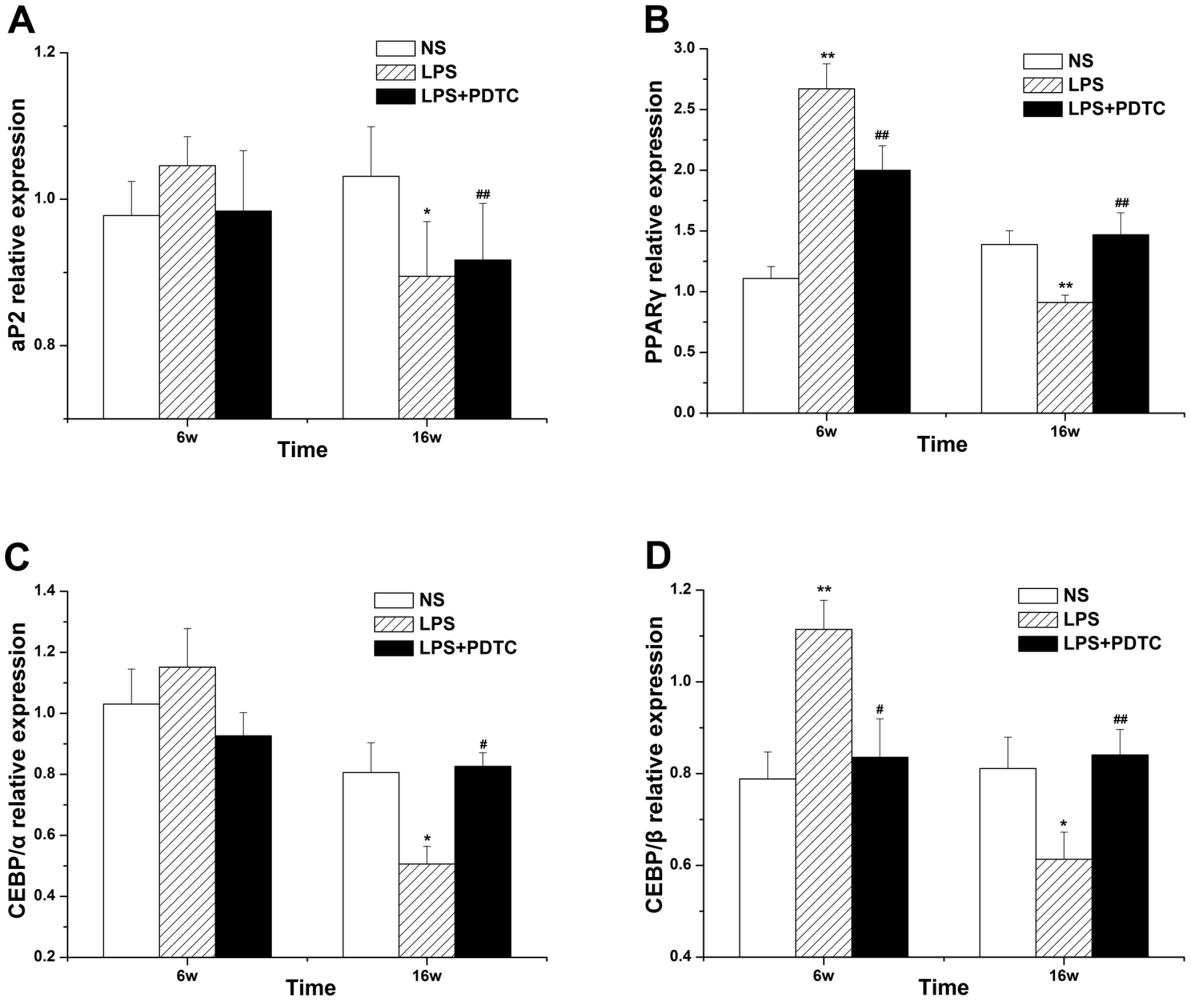
The effects of prenatal exposure to LPS/LPS+PDTC on aP2 (A), PPARγ (B), CEBP/α (C), and CEBP/β (D) expression in adipose tissue in offspring rats. n = 8. Values represent mean ± SD. *p<0.05, **p<0.01 vs. NS; #*p*<0.05 vs. LPS.

Compared with the NS group, the mRNA expression of PPARγ and CEBP/β in adipose tissue was significantly increased in the offspring of the LPS group at 6 weeks of age. PDTC treatment markedly inhibited the increase in PPARγ and CEBP/β mRNA expression. Moreover, aP2 and CEBP/α mRNA expression levels in the LPS group were higher than those in the NS group, and PDTC treatment reduced this change in mRNA expression, but the differences were not significant. In contrast, the expression of PPARγ, CEBP/β, aP2 and CEBP/α in the offspring of the LPS group was significantly decreased relative to the NS group at 16 weeks. PDTC treatment observably attenuated the LPS-induced decrease in the expression of PPARγ, CEBP/β, aP2 and CEBP/α in adipose tissue (Figure 7).

### Immunohistochemistry

**Figure 8:**
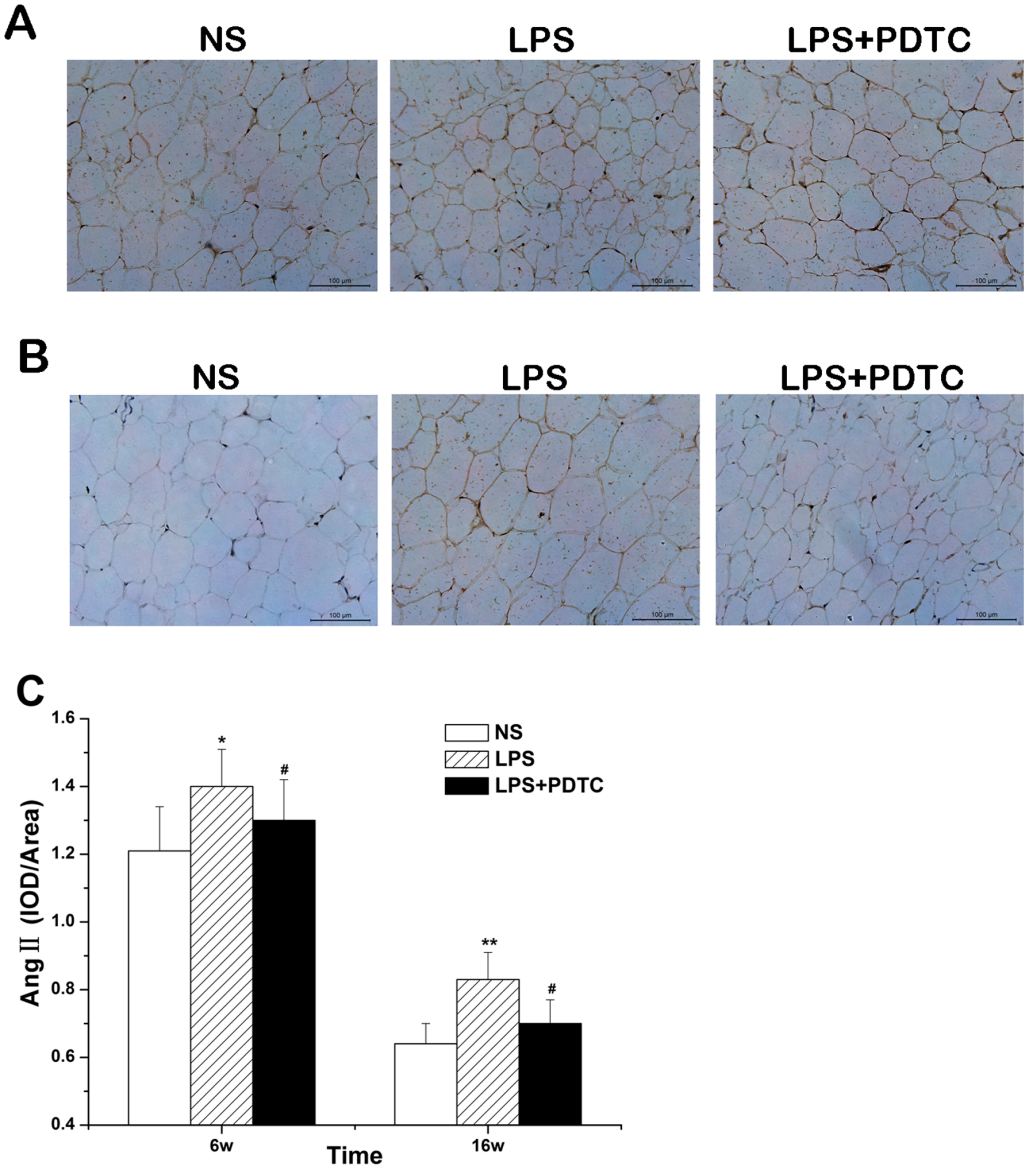
Prenatal exposure to LPS combined with PDTC alters Ang II expression in adipose tissue (immunohistochemistry) in 6- (A) and 16-week-old (B) offspring rats; Statistical analysis results (C). n = 8. Values represent mean ± SD. *p<0.05, **p<0.01 vs. NS; #*p*<0.05 vs. LPS.

The Ang II expression level in the offspring of the LPS group was significantly higher than that in the NS group at 6 and 16 weeks of age, with a more significant increase at 16 weeks of age (*P*<0.01). PDTC treatment markedly reduced the Ang II expression level (*P*<0.05) (Figure 8).

### Western blotting

**Figure 9:**
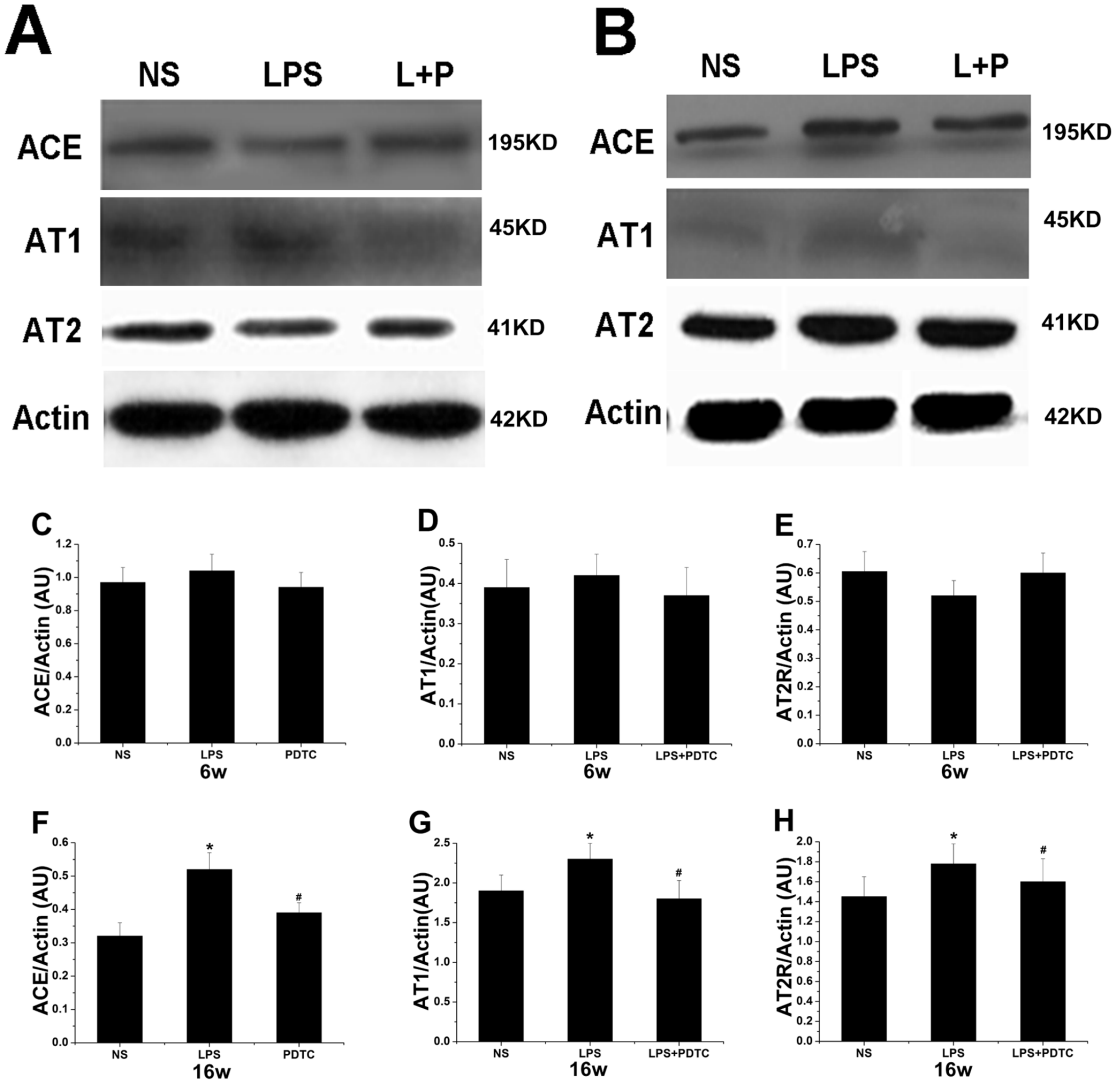
The effects of prenatal exposure to LPS/LPS+PDTC on ACE, AT1, and AT2 protein expression in adipose tissue in 6- (A) and 16-week-old (B) offspring rats. ACE expression at 6 weeks (C), AT1 expression at 6 weeks (D), AT2 expression at 6 weeks (E), ACE expression at 16 weeks (F), AT1 expression at 16 weeks (G), AT2 expression at 16 weeks (H). n = 6 per group. Values represent mean ± SD. *p<0.05, **p<0.01 vs. NS; #*p*<0.05 vs. LPS.

ACE and AT_1_R protein expression in the adipose tissue of the LPS group was higher than in the control group at 6 and 16 weeks of age, but it only reached significance at 16 weeks. PDTC treatment clearly reduced ACE protein expression at 16 weeks of age. Similarly, at 6 weeks, AT_2_R protein expression in the adipose tissue of the LPS group was lower than in the NS group, and PDTC treatment increased the expression level, but the differences were not significant. At 16 weeks of age, AT_2_R showed significantly increased expression in the offspring of the LPS group (*P*<0.05), and PDTC treatment mitigated the increase in AT_2_R protein expression (*P*<0.05) (Figure 9).

### mRNA expression of the local RAS in adipose tissue

**Figure 10:**
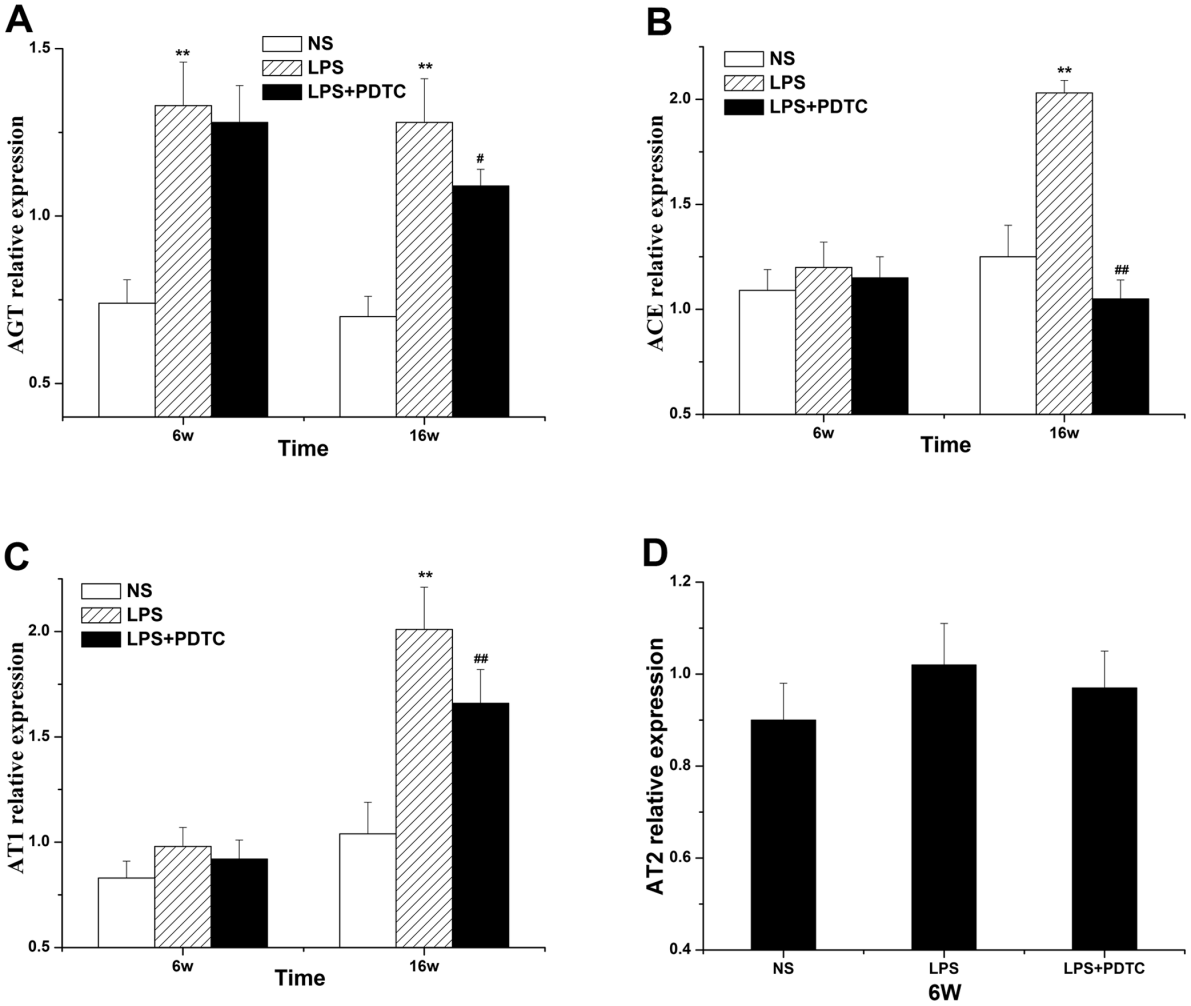
The effects of prenatal exposure to LPS/LPS+PDTC on local RAS mRNA expression in the adipose tissue of offspring rats. AGT at 6 and 16 weeks (A), ACE at 6 and 16 weeks (B), AT_1_ at 6 and 16 weeks (C), AT_2_ at 6 weeks (D). n = 6. Values represent mean ± SD. *p<0.05, **p<0.01 vs. NS; #*p*<0.05 vs. LPS.

Compared with the NS group, the expression in adipose tissue of mRNAs encoding AGT, ACE, AT_1_R and AT_2_R was increased in the offspring of the LPS group at 6 weeks of age but was only significant for AGT expression (*P*<0.01). PDTC treatment slightly inhibited the increase in AGT, ACE, AT_1_R and AT_2_R mRNA expression. At 16 weeks, AGT, ACE, and AT_1_R showed significantly increased expression in the offspring of the LPS group (*P*<0.01), and PDTC treatment observably inhibited the increase in AGT, ACE, and AT_1_R mRNA expression. In particular, the expression of AT_2_ in the offspring at 16 weeks was below the threshold of detection (Figure 10).

## Discussion

Hypertension is a chronic disease that not only damages the target organ but also reduces quality of life and shortens life expectancy; this condition requires lifelong treatment. According to recent studies, the occurrence of many adult chronic diseases is closely related to the growth environment of the fetus in utero [11], [12]. An animal model for prenatal exposure to LPS resulting in offspring rats with hypertension was successfully established after years of research in our laboratory. Using this animal model, our group conducted research on the cardiovascular, renal and neurological systems of offspring rats and found that prenatal exposure to inflammatory stimulation led to myocardial and vascular remodeling, damage to renal structure and function and cognitive defects in offspring rats [6], [13]. These results indicate that inflammatory stimulation during pregnancy is an important factor in the occurrence and development of hypertension in offspring.

In this study, we successfully duplicated the model of prenatal exposure to LPS (8th, 10th and 12th days of gestation) and found that prenatal exposure to LPS resulted in significantly increased blood pressure in offspring rats from 6 to 12 weeks. The body weights of the offspring of the LPS group were also significantly higher than those of the NS group after 2 weeks of age, and the LPS group offspring displayed increased adipose tissue wet weights and adipose coefficients. The fat cells of offspring rats were observed under 40× magnification, which showed that the stimulus led to an increased number of fat cells but decreased fat cell diameter in juvenile offspring rats. Conversely, the stimulus induced larger adipocytes and fat deposits in adult offspring rats. Moreover, the glucose AUC in 16-week-old rat offspring was strikingly increased in the LPS group compared with the NS and LPS+PDTC groups. The OGTT results indicated that prenatal exposure to LPS resulted in abnormal glucose tolerance in offspring rats. However, the lipid levels exhibited no significant differences. PPARγ is the transcription factor for the process of adipocyte differentiation, and it is regarded as a marker of adipocyte differentiation along with CEBP and aP2. The results suggest that prenatal exposure to LPS resulted in increased adipocyte differentiation in juvenile offspring rats but inhibited adipocyte differentiation in adult offspring rats. PDTC treatment observably improved the LPS-induced anomalies in body weight, blood pressure and fat development.

The results mentioned above indicate that prenatal exposure to LPS resulted in increased blood pressure, adipose wet weight, adipose coefficient and body weight in offspring rats. Specifically, the stimulation caused fat abnormalities characterized by changes in fatty cell morphology and adipocyte differentiation. However, PDTC can reverse the influence of the LPS stimulus during pregnancy. In fact, research has shown that captopril (angiotensin-converting enzyme inhibitors) can reduce the weight of C57BL/6J mice fed with high-fat diets and improve glucose tolerance, whereas fat cells become smaller [14]. Moreover, irbesartan (Angiotensin II type I receptor blockers) can increase the activity of PPARγ to promote adipocyte differentiation, increase the number of adipocytes and improve the dysplastic adipose tissue [15]. Taking the literature into account, we speculate that the mechanism by which prenatal exposure to inflammatory stimulation affects blood pressure in offspring may be related to the abnormal development of fat.

The RAS is an important humoral system consisting of renin, angiotensin and its receptor. The RAS can adjust the normal physiological function of the cardiovascular system and is also involved in the pathological processes of hypertension, myocardial hypertrophy, congestive heart failure and other diseases [16]. AGT is the source of RAS components, and it plays a key role in the production of Ang II. Ang II is an important and active constituent of the RAS; it has different functions in different tissues, but the final result in every tissue is vasoconstriction and increased blood pressure [17]. It exerts its biological effects by binding its specific receptors AT1 and AT2 [18]–[20]. The AT1 receptor is mainly distributed in the blood vessels, heart, liver, brain, and kidney. In mature tissues, the effects of Ang II are all mediated by the AT1 receptor [21]. These effects include promoting protein phosphorylation, facilitating cell proliferation, accelerating the contraction of vascular smooth muscle and controlling the intake of water and the excretion of urinary sodium [22]. The AT2 receptor is highly expressed in the blood vessels during embryonic development, but it drops rapidly after birth and becomes mainly distributed in myocardial tissue, vascular smooth muscle [23], endothelial cells and nerve fibers around the blood vessels. The main functions of AT2 are promoting protein phosphorylation, inhibiting cell proliferation, dilating the blood vessels, and promoting diuresis [24].

There is a complete RAS in adipose tissue that does not rely on the circulatory system. The mRNA expression levels of AGT, ACE, AT_1_ and AT_2_ in adipose tissue were examined by RT-PCR. The results showed that the mRNA expression of adipose tissue AGT in the offspring of the LPS group increased significantly at 6 and 16 weeks of age and that the expression of adipose tissue ACE, AT_1_ and AT_2_ in the LPS group increased at 6 and 16 weeks but was only significant at 16 weeks. The expression of AT_2_ in the offspring at 16 weeks was too low to be detected. However, PDTC treatment alleviated the aberrant LPS-induced expression of RAS genes. Meanwhile, when RAS activity was measured at the protein expression level, the results showed that prenatal exposure to LPS significantly increased the protein expression level, thereby implying that the RAS was activated.

In conclusion, inflammation induced by prenatal exposure to LPS results in increased body weight, adipose coefficient and blood pressure that might develop into hypertension in adult rats. During the juvenile phase in offspring rats, prenatal stimulation activates local adipose tissue RAS to promote adipocyte differentiation (yielding a smaller diameter and area of adipocytes but increasing the quantity of adipocytes). Conversely, in adult offspring rats, excessive prenatal stimulation activated local adipose tissue RAS, thus inhibiting the differentiation of adipocytes (yielding a larger diameter and area of adipocytes and resulting in lipid accumulation). At the same time, PDTC reversed the LPS-induced dysplasia of adipocytes in offspring rats that produces offspring with increased blood pressure and activated local adipose tissue RAS. Prenatal exposure to inflammatory stimulation results in increased blood pressure and abnormal adipocyte development; we infer that these results are strongly associated with the activated state of local adipose tissue RAS in offspring rats. These results are relevant in that anomalous local adipose tissue RAS and adipose development may be an important mechanism underlying hypertension.
